# Waves on Louisiana Continental Shelf Influenced by Atmospheric Fronts

**DOI:** 10.1038/s41598-019-55578-w

**Published:** 2020-01-14

**Authors:** Biyun Guo, M. V. Subrahmanyam, C. Li

**Affiliations:** 1 Marine Science and Technology College, Zhejiang Ocean University, Zhoushan, Zhejiang 316022 P.R. China; 2 State Key Laboratory of Plateau Ecology and Agriculture, Qinghai University, Xining, Qinghai 810016 P.R. China; 3 Department of Oceanography and Coastal Sciences, Louisiana State University, Baton Rouge, Louisiana 70803 USA

**Keywords:** Physical oceanography, Environmental sciences, Ocean sciences

## Abstract

Ocean-atmospheric dynamical processes influence the wave characteristics, and sea surface temperature (SST). The processes that affect SST in the ocean area included surface heat fluxes, wind, and precipitation. In this study, we analyzed the wave data in response to the cold front passages over Louisiana continental shelf. The data examined in this research is mainly from WAVCIS (Wave-Current-surge Information System), Coastal Ocean Estuarine Dynamics Lab at Louisiana State University and Global Precipitation Climatology Project (GPCP). With respect to the wave response to the atmospheric forcing, here we consider: (1) the connection between wave variability and atmospheric frontal passages, and (2) the influence on the SST variations during the processes. The occurrences of wind wave, precipitation, and weather processes have distinct regularities. Atmospheric cold fronts have an important influence on wave formation and associated processes over Louisiana continental shelf.

## Introduction

The Louisiana Continental Shelf along the north-central Gulf of Mexico, including the inner continental  shelf, lower plain, estuaries, bays and barrier  islands, contains 40% of the U.S. estuarine wetlands. This area is one of America’s most important coastal ecosystems in terms of oil production infrastructure, natural resources, and cultural heritage. This region also faces a serious environmental problem of having the highest rates of wetland loss and coastal erosion in the nation due to the combined effects of anthropogenic activities and natural processes during the past century^1^. The Louisiana coast has a significant  variability of wind pattern at different spatial and temporal scales. In the Gulf of Mexico, cold fronts dominate between Oct. and April, affecting the coastal sediment transport and wetland variations. The coastal wetlands have been eroding at an alarming rate since past century, and the deltaic landscape has changed dramatically^2–4^. Although the northern Gulf of Mexico is located in a relatively low tidal and wave energy zone, the trend of the sedimentation and erosion in the low-lying plains of Louisiana Delta are strongly affected by coastal processes, of which subsidence and the effects of frequent winter-spring cold fronts and occasional tropical cyclones are significant^5^. A large number of ocean phenomena are associated with the passage of cold fronts, when temperature and humidity decrease significantly while wind switches to relatively strong northerly, resulting in flushing of bays, and sediment resuspension and transport^6–9^. Wind-driven coastal surge is an important contributor to the regional flooding, although high water levels are  also influenced by tides, riverine currents, atmospheric pressure, wave and rainfall. The strength of the pneumatic system, the speed of advance and the frontal orientation relative to the shoreline, will control the coastal processes^10^.

The Louisiana coast is vulnerable to developing larger storm surge because of the local geographic configuration. Particularly, the barrier islands, extensive manmade levees, low-lying topography, large interconnected shallow lakes, and a protruding delta on the Louisiana-Mississippi-Alabama shelf characterize the east bank of Mississippi River in the southeastern of Louisiana. These geomorphologic characteristics can amplify the surge as water is blown from both the south and east to the continental shelf and then blocked by the riverbanks, delta, and barrier islands. An extensive low-lying wetlands, large inland lakes, and east-west coastline characterize the west of Louisiana. These features tend to reduce surge heights due to the southerly winds only in the right center quadrant of the storm push water against the Louisiana coast effectively, and the low-lying wetlands can weaken transient surges in this zone^11^. Sediment diversion to the seaward receiving basin provides more surge protection due to active coastal processes, however sediment retention rates tend to be lower^12–17^.

Louisiana is experiencing severe wetland degradation, loss, and related ecosystem problems^18–20^. The problem of coastal land loss has become disastrous with more than 100 km^2^ per year over the last century^1^. Coastal erosion in Louisiana has been studied through examining the relationship between hydrodynamic, sediment transport trends, and meteorology process. Recent work has also included the application of numerical models for the hydrodynamic process and mechanisms, sediment transport, and morphological changes of Louisiana Continental Shelf in different weather conditions. Water level fluctuation varies greatly in spatial and temporal scales, subject to influence from tides, river flow, vegetation, climate change, atmospheric forcing, and anthropogenic activities^21–26^.

In the Gulf basin, tidal forces tend to dominate circulation, but local winds are also important to control the general low-frequency (or subtidal) flow direction^18,19^. Although cold fronts are less severe than hurricanes and the related wind surges are much smaller than a major storm surge, they often occur in winter and spring: the time interval between fronts is about 3–7 days, which makes a total of 20–30 events every winter and spring^27,28^.

In the bay/estuarine environment along the northern Gulf, cold fronts play an important role in the morphological evolution of the dune-beach system on various time scales from annual to decadal. Many studies have shown the relationship between coastal waves, currents, sediment concentration and erosion/deposition pattern during the period of frontal passages^29–32^. These efforts will help future predictions of continental shelf evolution and better regional recovery strategies. For wetland restoration and coastal management, it is necessary and critical to know how hydrodynamic processes, sediment transport, meteorological parameters are functioning together in such unique coastal geomorphologic and hydrological system. Studies have shown that, wave plays an important role in coastal erosion by wave-sediment interactions, wind-driven and tidally-driven effects. Stone *et al*.^33^ pointed out that since 1901, there were 55 hurricanes or tropical storms landed along the Louisiana coast, and these storms may account for an estimated 90% of barrier islands coastline retreat by the inlet breaching and over wash processes. However, they suggested that sediments could also deposit in coastal wetlands, which could offset ongoing wetland loss.

A cold front is the interface or transition zone (25–250 km) between heterogeneous air masses where cold, dry and denser air is advancing towards warmer, moister and lighter air^34,35^. Cold front passages are the dominant local weather patterns, along the Gulf coast of the United States between October and April^35,36^. In the Atchafalaya Delta, water bodies and active waves affected by the frontal wind erode continental lobes, resuspend and transport bottom sediment to saltmarsh and in the Chenier coast without marine fluid slurry severely eroded by wave action. Post frontal wind causes the water level to drop rapidly and streams the turbid bay water onto the shelf. Barrier islands are strongly eroded by the waves produced by the long- fetch prefrontal winds, and the continuous land wind could cause wind transport and dune migration. The post frontal winds reshape landward ends of wash over lobes, and deflect the surface of the lobes through wind actions^10^.

In this study, we analyze wind and wave time series, and the timing of cold front passages measured at the area (89°–91°W, 28.5°–29.5°N) from 1st to 30th April, 2010. We examine the wave variation relationship with the cold fronts and associated wind, and precipitation.

### Study site

**Figure 1:**
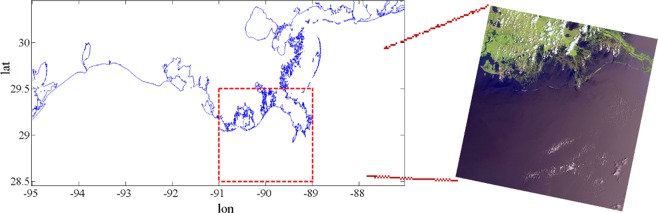
Location map of the study area along with Remote Sensing image presenting the ocean-land pattern in space. The figure generated by using ArcGIS software.

The study area is over Louisiana continental shelf (Fig. 1), from east to west of Louisiana shelf, Barataria Bay, Timbalier-Terrebonne Bays, and Atchafalaya-Vermilion Bays. The characteristics of these bays are show in Table 1.

**Table 1:** Characteristics of bays along Louisiana coast (modified from USEPA, 1999).

Content	The name of the bay		
	Timbalier-Terrebonne Bays	Barataria Bay	Atchafalaya-Vemilion Bays
Drainage area (km^2^)	4100	5700	260600
Surface area(km^2^)	1761	1673	1821
Average depth(m)	2	2	2
Coastal wetlands(km^2^)	1020	—	1870
Average salinity(psu)	18	13	1
Average daily freshwater inflow(m^3^/s)	130	156	6337

Timbalier-Terrebonne Bays are wide and shallow estuaries that receive fresh water from the surrounding estuaries and canals. Although the two bays are interconnected, they belong to two different barrier systems. Timbalier Bay belongs to Bayou Lafourche barrier system and Terrebonne Bay is part of the Dernieres Isle system^37^. Barataria Bay, about 19 km wide and 24 km long, in southeastern Louisiana, its entrance is largely blocked by Grand Terre Islands and Grand Isle is via a narrow Gulf channel navigable through connecting waterways to the Gulf intracoastal water system. Because of wetland loss and submergence, Barataria Bay is continuously increasing in depth and size, inducing to an increase of tidal current velocity, water storage volume, inlet cross-sectional area, and sediment storage capacity^37^. The Atchafalaya-Vermilion Bay system is comprised of five inter-connected coastal bays affected by wind forcing and seasonally varying river inputs. Atchafalaya River discharges approximately 30% of the Mississippi River flow and 30–40% of the Mississippi River sediment load into the coastal ocean with a peak discharge usually in spring through the Wax Lake Outlet and Atchafalaya River^6^.

### Data

The oceanographic data are available from the Global Precipitation Climatology Project (GPCP) datasets over the study area (89°–91°W, 28.5°–29.5°N). The GPCP V.1.3 daily precipitation (mm/day) with a time resolution of 24 hours, daily relative humidity (%) at surface, and daily mean surface pressure (pascal) are obtained from NCEP, and NOAA optimum interpolation daily sea surface temperature (°C) with 1/4 degree resolution for the period of 1st to 30 April 2010. In addition, 6-hourly air temperature (°C) from 1st to 30th April 2010 used from MERRA-2 Version 5.12.4. Moreover, measured data include wave height, wave period, water level, wind speed, and wind direction from the WAVCIS station (CIS 6) (http://www.wavcis.lsu.edu/). The ADCP installed at the bottom of WAVCIS provides current data, which can be analyzed for the effects of different factors. Measurements of mean surface pressure, relative humidity, and precipitation, sea surface temperature (SST), air temperature, wave height, wave period, wind speed, wind direction are given in Table 2.

**Table 2:** The data information used in this study.

Data set	Variable	Unit	Sampling interval	Number of data
GPCP One-degree daily precipitation, V.1.3	Precipitation	mm/day	24 hours	30
NCEP: Daily relative humidity on pressure levels	Relative humidity	%	24 hours	30
NOAA optimum interpolation 1/4 degree daily SST blended with AVHRR and AMSR data version 2	SST	°C	24 hours	30
NCEP: Daily mean surface pressure	Surface pressure	pascal	24 hours	30
MERRA-2 instantaneous, analyzed meteorological fields on pressure levels version 5.12.4	Air temperature	°C	6 hours	244
WAVCIS station, CIS 6 (http://www.wavcis.lsu.edu/)	Wave height	m	hourly	720
	Wave period	s	hourly	720
	Wind speed	m/s	hourly	720
	Wind direction	degree	hourly	720

## Results

### Variation of atmospheric parameters and wave

**Figure 2:**
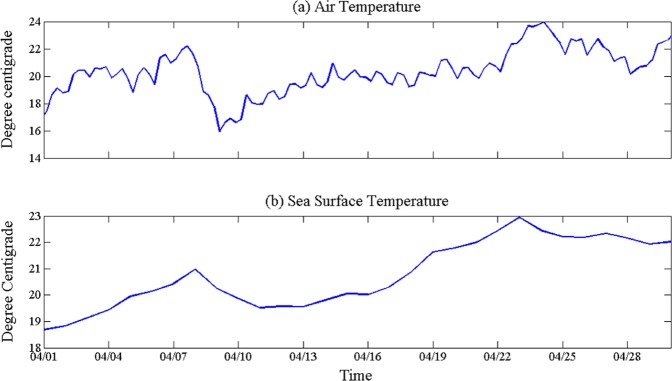
Air temperature (MERRA-2, version 5.12.4) and SST (NOAA optimum interpolation daily SST data version (2) variation in April 2010 over Louisiana Continental Shelf.

We have analyzed the wave and wind and other atmospheric parameters from 1–30 April 2010, to establish the relations among them. Figure 2 shows the variations of air temperature and SST respectively during the study period. Air temperature shows the daily fluctuating and the mean value increases from 17 °C to 23 °C in April 2010, and it dropped sharply about 5 °C from 7 to 9 April. The daily SST varied between 18 °C and 23 °C, with an increasing trend similar to that of air temperature.

**Figure 3:**
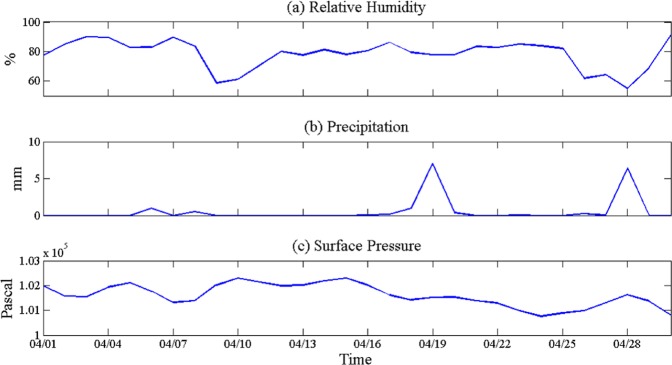
Relative humidity (from NCEP daily data), precipitation (from GPCP daily data), surface pressure (from NCEP daily data) variations plotted for the April 2010 month over Louisiana Continental Shelf.

Figure 3 depicts the daily variations of relative humidity, precipitation and sea level pressure during April 2010 in the study area. The relative humidity fluctuates greatly (Fig. 3a), the maximum value is 91%, the minimum value is 54.75%. Figure 3b shows the daily precipitation. There were five rainfall events in April, which were 6, 8, 18, 19 and 28th, and the highest rainfall occurred during 19 and 28th, and the precipitation are 7.053 and 6.398 mm respectively. Daily mean surface pressure are given in Fig. 3c, there were three low pressures (3, 7, and 24) and four high pressure (5, 10, 15, and 28) during April 2010, with the values of 101550, 101320, and 100760 Pascal; and 102110, 102290, 102300, and 101630 Pascal respectively.

### Influence of atmospheric fronts on weather

Fronts may be classified into three categories (cold, warm, and stationary fronts). The leading edge of a forward moving cold air mass is the cold front. The edge of the advancing warm air mass is the warm front – as the transition zone where a warm air mass is replacing cold air mass. When neither air mass advances toward another air mass, the front is stationary^38^.

 The cold front moves at a velocity of the wind component perpendicular to the front just above the frictional layer. Cold front frequently occurs in the northern Gulf of Mexico. The passage of cold front affects the main local weather pattern^39–41^. Many meteorological and oceanic phenomena are associated with cold front passages, such as wind and current variations, significant decrease in humidity and temperature, water level variation, flushing of bays^9^, and resuspension of sediment and transport of water and sediment^42–45^. Due to different configurations of the cold front, the distribution of cloud and precipitation near the cold front is also obviously different, some mainly occur behind the front, others mainly in front. When the cold frontal passes, it is accompanied by an increase in northerly wind, air pressure rising and temperature decreasing. Sometimes it comes with rain or snow, and even heavy rain. In general, after the cold front passes, the local area will be controlled by cold high pressure and the weather will become sunny. While cold front is moving, the humidity reduced, and the sea level pressure increases. It is clearly understood (Fig. 3) that, when cold front moves over the area, it brings in the high latitude dry air, which reduces the humidity in the region. For example, from 7 to 9th, the temperature was lowered and the relative humidity also reduced, the same trend occurred from 25 to 28th. The sea level pressure increases due to cold air passing through the area.

**Figure 4:**
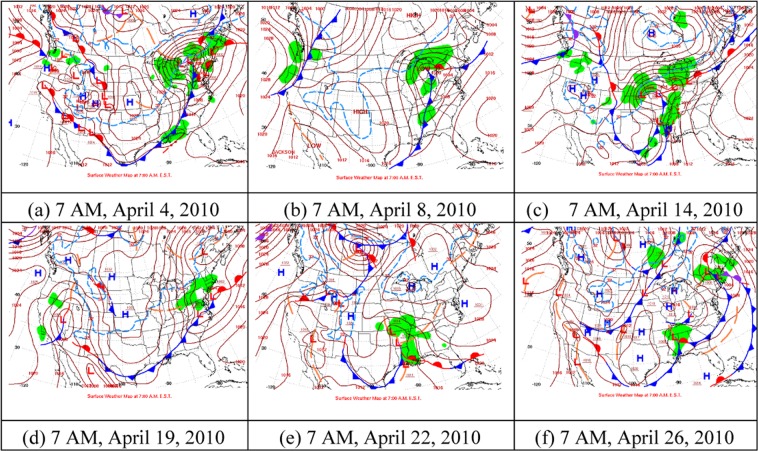
Surface weather maps corresponding to the six frontal passages events. Cold fronts are in blue lines with triangles on the warm side of the front. Low pressure and high pressure areas are L and H respectively. Green patches are the rainfall. (**a**) Surface weather map on 7AM, April 4, 2010, (**b**) surface weather map on 7AM, April 8, 2010, (**c**) surface weather map on 7AM, April 14, 2010, (**d**) surface weather map on 7AM, April 19, 2010, (**e**) surface weather map on 7AM, April 22, 2010 and (**f**) surface weather map on 7AM, April 26, 2010. All the weather maps given above are from the National Centers for Environmental Prediction, Weather Prediction Center. (https://www.wpc.ncep.noaa.gov/dailywxmap/).

Figure 4 shows the weather maps of the frontal passage events in April 2010. The maps are from the National Centers for Environmental Prediction, Weather Prediction Center. (https://www.wpc.ncep.noaa.gov/dailywxmap/). There are 6 cold fronts, which are on 4, 8, 14, 19, 22, and 26th, of which 8th April front passage is dominant (see Fig. 4). When the cold frontal passed, the temperature dropped on the 9th, which was reduced by 8 °C from 8 to 9th, while the other several events did not have effect on the temperature significantly. In addition, before the air temperature dropped rapidly, it increased approximately 2 °C within 2 days. The air temperature was reduced 3 °C from 24 to 27th, which lasted 3 days.

This change process of air temperature is associated with the cold-front passage as the prefrontal southerly winds blow over warmer air and the postfrontal northerly winds are usually much colder. Before the cold front passage, it is controlled by low-pressure warm air mass, it is low air pressure, and high temperature. When the cold front passes, the cold air mass quickly squeezed and cut down the warmer mass. Due to the intense extrusion of the cold and warmer air mass, strong convective weather such as heavy rain and strong wind will occur (Fig. 4), and the temperature will drop rapidly in the study area.

From Fig. 4, it is evident that the cold front is moving over the Louisiana coast. Due to the cold front, air temperature decreased rapidly leading to the reduction in SST (Fig. 2). The high-pressure system also moves along with the cold front. On 9th the high pressure system split into two, but the Louisiana coastal area was occupied by a high pressure system, which later moved to the east.

**Figure 5:**
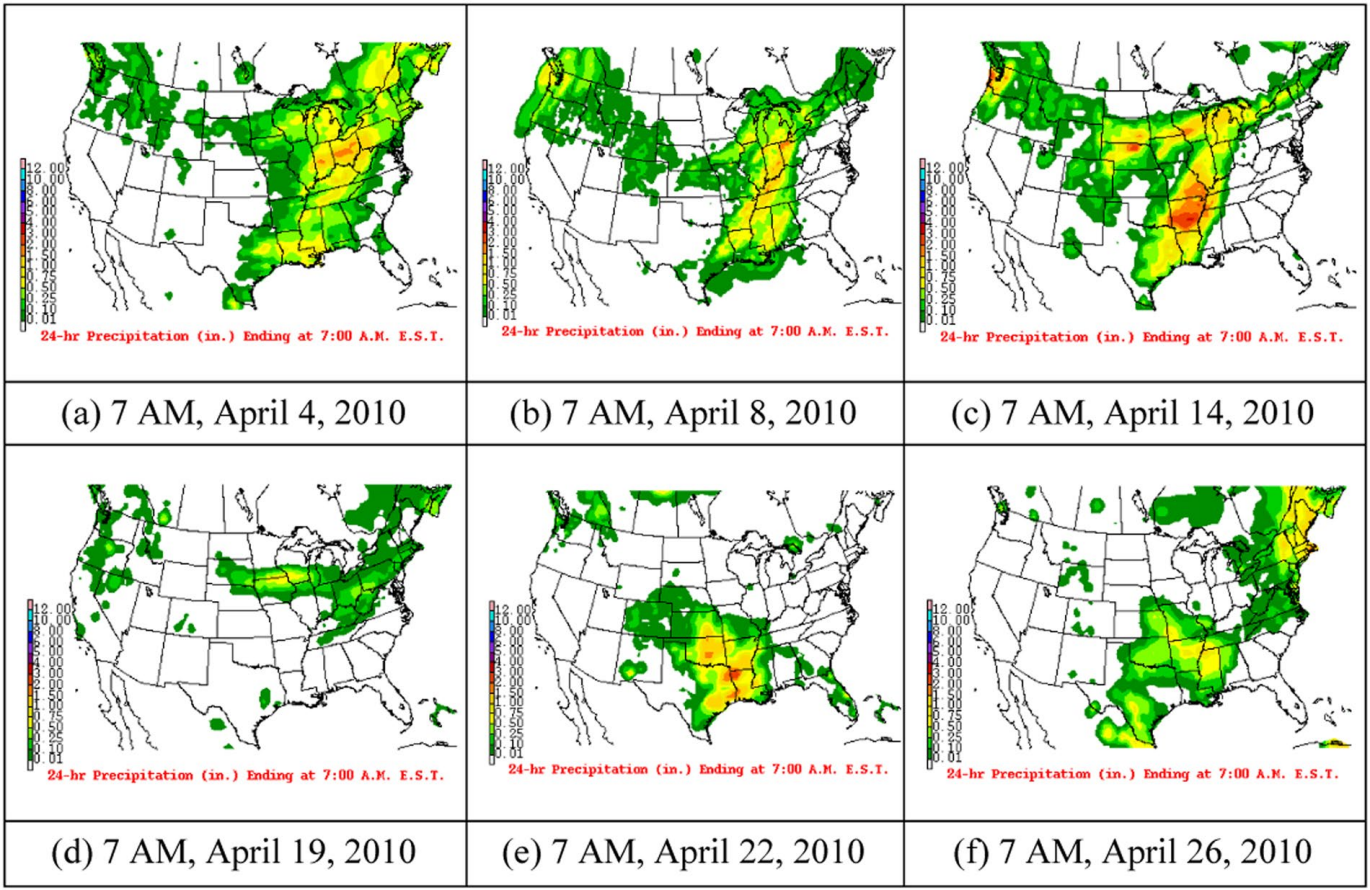
Precipitation patterns corresponding to the six frontal passages events. (**a**) On 7 AM, April 4, 2010, (**b**) on 7 AM, April 8, 2010, (**c**) on 7 AM, April 14, 2010, (**d**) on 7 AM, April 19, 2010, (**e**) on 7 AM, April 22, 2010 and (**f**) on 7 AM, April 26, 2010. All the weather maps given above are from the National Centers for Environmental Prediction, Weather Prediction Center. (https://www.wpc.ncep.noaa.gov/dailywxmap/).

Figure 5 is the precipitation patterns corresponding to the six frontal events. From Fig. 5, we can see that, when the front moves, the rain band also moves with the front. On April 4, 8,14,19,22, and 26th Louisiana coast experienced the precipitation, which also affected SST.

The land-sea breeze effect is evident along the northern Gulf in summer. In winter and spring, however, the majority of the weather variability is caused by the wind associated with cold fronts or cold air outbreaks. According to the analysis of wind data, the front usually has the southwest-northeast orientation and plays an important role in controlling the coastal processes^9^, giving the east-west orientation of the northern Gulf of Mexico coast^46^.

**Figure 6:**
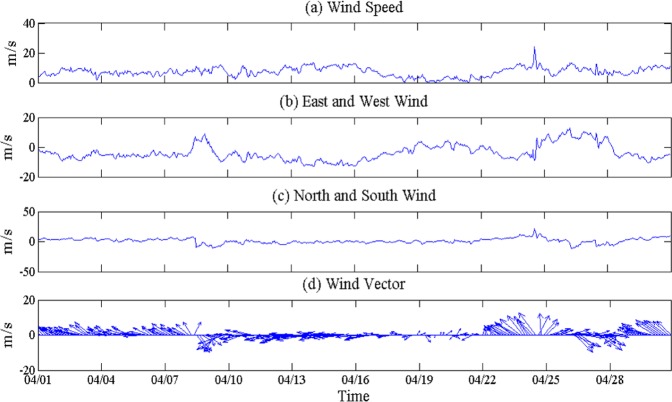
The time series of wind speed and direction taken from the Wave-Current-Surge Information System (WAVCIS) during April 2010. (**a**) the wind speed, (**b**) the Zonal wind, (**c**) Meridional wind and (**d**) represents the vector representation of wind speed with direction. (**d**) Clearly indicating the wind pattern over the Lousiana bay during April 2010.

Wind data show that the southerly and northerly wind account for ~38% and 62%, respectively. The wind speed and direction of cold frontal events in April are given in Table 3. Figure 6 illustrates the time series of wind speed and direction. Generally, when the wind suddenly switched to the north wind, it indicates that the cold front passage of the study area. This is verified by the atmospheric pressure, air temperature, and weather maps. Ahead of these northerly winds, the south winds are dominant. The winds also appears to have clockwise rotation, similar to inertial oscillation^47,48^. From Figs. 4 and 6, we can see that the wind variations is related to fronts. With respect to frontal movement, wind also changes its direction.

**Table 3:** The wind speed and direction of cold front during April 2010.

Data	April 4	April 8	April 14	April 19	April 22	April 26
Wind speed(m/s)	6.40	8.15	10.41	4.10	5.22	10.44
Wind direction(°)	113.17(SE)	239.88(SW)	85.67(NE)	203.04(SW)	157.25(SE)	295.04(NW)

*SE, southeast; SW, southwest; NE, northeast; SW, southwest; NW, northwest.

**Figure 7:**
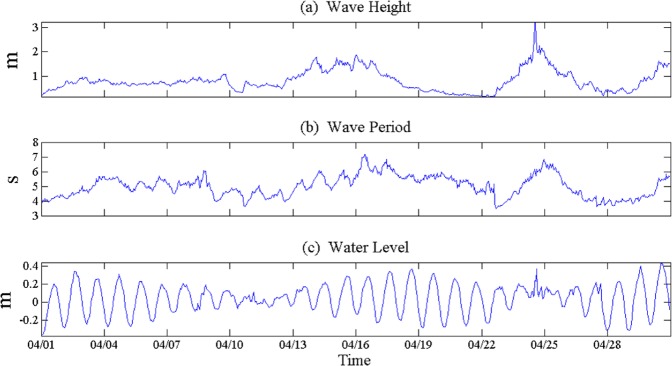
The time series of wave and water level from the Wave-Current-Surge Information System (WAVCIS) during April 2010. (**a**) Wave height, (**b**) wave period and(**c**) water level variations.

The cold fronts play an important role in the short-term evolution of the Northern Gulf coastal bays and estuaries. The pre-frontal wind blows from the south, producing strong waves along the Gulf-facing beaches. The post frontal wind blows from the north then generates high-frequency and high-energy waves in the bays and adjacent estuaries, where the fetch is long enough^46^. Data used from different sources clearly demonstrate the frontal effects on the waves. In Fig. 7, time series of wave height, wave period, and water level are presented for April 2010.

Figure 7 shows wave height, wave period and water level, the data interval is hourly. From Fig. 7, when the cold front passed, wind speed was at the low, water level also decreased, with a several-hour delay. Wind directions were northern (NE and NW) and the wind speed was larger than other events on 14 and 26th, and the wind directions in the other events were southerly winds (SE and SW). Although the water level was at its low, it was higher than other events in these two days (14 and 26), which is related to the northerly wind. When the northern wind is blowing, wind stress causes the water level to drop.

Before the arrival of each event, winds are from the south, showing a continuous switch to the west and north as the frontal boundary gets closer to the coast (Figs. 4 and 6). The northerly wind associated with the post-frontal phase generally lasts for 1–2 days, slowing down and switching to the south. The wave heights of each event (4, 8, 14, 19, 22, and 26th) are 0.72 m, 0.84 m, 1.36 m, 0.4 m, 0.26 m, and 0.82 m respectively, and are consistent with peaks in continuous wind speed. Wind and wave directions have a strong correlation, since the peak wind energy should be closely related to the peak wind speed. The northerly wind blew from 9 to 11th, the wave height decreased as the wind speed decreased, since wind fetch was small. It was southerly wind from 22 to 25th, wind speed, wave height and wave period increased, due to the longer fetch.

## Discussion

The Louisiana coast experiences high erosion rates over the last few decades. In this study, we discussed  a few parameters influenced by atmospheric fronts in Louisiana Continental Shelf. The majority of weather conditions experienced in Louisiana coast are due to the active cold fronts. The movement of the frontal systems through this region in winter depends on the large scale atmospheric circulations and air mass trajectory behind the front. The cold front may be associated with the development of wave on the quasi-stationary front system. The waves can propagate in different directions off Louisiana coast, as the cold fronts moving through the region. The weather associated with frontal passage can be accompanied by significant precipitations and thunderstorms at times.

As we discussed earlier that cold fronts are frequent in winter and spring, and the time interval is about 3–7 days between fronts^9^, which makes 20–30 events from each winter to the following spring^49^. The variability of different cold front passage events is large in April 2010. The variation in wind direction during cold front passage was found to have a significant impact on the wave height and water level. The north wind is primarily after the passage of the front along the coastal Louisiana continental shelf. The southerly moving cold fronts produce strong winds (onshore wind) by which the higher amplitude waves are produced and water setup over the coast^9^. After the water setup over the coast, wind changes rapidly from the north (offshore wind) leading to water level set down, which drains the shallow bay area^9,28^. The associated water setup and set down during frontal passage, when the wind quickly switched from south to north, have significant implications to Louisiana’s bays and coastlines. The wind-driven oscillation of the shallow bays can have a significant impact to the coastal region. The water level change induced by cold front can be on the order of 1 m and associated flow velocity can reach more than 0.5 m/s^9,28^. When cold front prevails the heat exchange happens between atmosphere and ocean (sensible heat flux) and when the higher wind prevails the latent heat flux will be higher. When latent heat flux is higher, humidity will be higher in the atmosphere.

The hydrodynamics can significantly influence the sediment transport^50^. Before the cold front passage, the effect of southerly winds and subsequent wave and water level setup typically causes sediment transport to the nearshore, which might result in deposition. In the period of post-frontal (northerly) winds, water level drops and the suspended sediment moves out. However, during the subsequent front events, when sediment is revised from a shallow nearshore, the resuspension of sediment and deposition on the shallow water can be accomplished. As a result of the inherent dynamical process in cold front passages over the northern Gulf of Mexico, a huge turbid coastal plume can form, extending 180 km alongshore and 75 km offshore away from the Louisiana coastal^51^.

## Conclusion

In this study we examined the relationship among the changes in wave height and sea surface temperature and the air-sea interaction parameters such as cold front, wind, SST, precipitation etc. The findings are as follows: the cold front passed through the coastal area on the 8th of April, reduced the SST by 5 °C. The wind direction changed from SE to NW. The wind and wave appeared to have a strong relation. The cold front caused the water temperature to decrease in the study area as the air pressure increased, while the wind changed to northwesterly, the wave height increased then decreased and the wave period decreased then increased. Because of the cold front, wind changed its direction, and the precipitation occurred, all of which led to the decrease of SST.

 Several cold front events occurred in April 2010, but they were not strong. There were two events occurred during 22–26th, in which the wind direction varied from SE to SW, during the same period the wave height increased rapidly. The direction of wave and wind are similar resulting an increase in wave height, however the wave period increased with a lag of 1 day. Cold frontal events are also associated with the changes in sea level pressure and humidity.
